# German brass for Benin Bronzes: Geochemical analysis insights into the early Atlantic trade

**DOI:** 10.1371/journal.pone.0283415

**Published:** 2023-04-05

**Authors:** Tobias B. Skowronek, Christopher R. DeCorse, Rolf Denk, Stefan D. Birr, Sean Kingsley, Gregory D. Cook, Ana María Benito Dominguez, Brandon Clifford, Andrew Barker, José Suárez Otero, Vicente Caramés Moreira, Michael Bode, Moritz Jansen, Daniel Scholes

**Affiliations:** 1 Department of Machine Engineering and Material Sciences, Technische Hochschule Georg Agricola, Bochum, Germany; 2 Department of Anthropology, Maxwell School of Citizenship and Public Affairs, Syracuse University, Syracuse, New York, United States of America; 3 Eucoprimo, [European Union to Search for, Collect, and Preserve Primitive and Curious Money], Rüsselsheim, Germany; 4 Zalando SE, Berlin, Germany; 5 Wreckwatch Int., London, United Kingdom; 6 Department of Anthropology, University of West Florida, Pensacola, Florida, United States of America; 7 Sociedad de Ciencias Aranzadi, San Sebastian, Spain; 8 Whydah Pirate Museum, Yarmouth, Massachusetts, United States of America; 9 Department of Geography, University of Santiago de Compostela, Santiago de Compostela, Spain; 10 Museo do Mar de Galicia, Vigo, Spain; 11 Deutsches Bergbau-Museum, Bochum, Germany; 12 Shipwreck Treasure Museum, Charlestown, United Kingdom; Universita degli Studi di Milano, ITALY

## Abstract

Utilizing geochemical analysis, this study identifies the sources of European brass used in the casting of the renowned Benin Bronzes, produced by the Edo people of Nigeria. It is commonly believed that distinctive brass rings known as “manillas”, used as currency in the European trade in West Africa, also served as a metal source for the making of the Bronzes. However, prior to the current study, no research had conclusively connected the Benin artworks and the European manillas. For this research, manillas from shipwrecks in African, American and European waters dating between the 16th and 19th Century were analysed using ICP-MS analysis. Comparing trace elements and lead isotope ratios of manillas and Benin Bronzes identifies Germany as the principal source of the manillas used in the West African trade between the 15^th^ and 18^th^ centuries before British industries took over the brass trade in the late 18^th^ century.

## Introduction

The Benin Bronzes collectively refers to thousands of African artworks in the form of heads, plaques, figurines and other objects dating between the 16th and 19th Century AD. Although replicas exist [1] the vast majority of Benin pieces in European and United States museums derive from the infamous British expedition of 1897 [2]. The Benin material has been subject to varied geochemical analyses. However, while clearly of African manufacture, the dating of the artworks and the origin of the metal used in their casting has remained unknown. The current study demonstrates that the primary source of the brass used in the Benin bronzes was produced in Germany and was likely shipped to West Africa in the form of horseshoe-shaped brass rings, known as manillas.

Over 700 available chemical analyses of Benin Bronzes [3] indicate that the majority are either brass or leaded brass, sometimes with small amounts of tin. Their chemical and lead isotope composition is much different from earlier Nigerian bronzes such as those from Igbo-Ukwu [4, 5]. Documentary sources indicate that hundreds of thousands of open horseshoe-shaped copper and brass rings, known as manillas, were shipped from Europe to West Africa with the opening of the Portuguese trade in the late 15th century, and it has been suggested that they were the metallic source of the Benin Bronzes [6–10]. However, previous studies comparing available manillas to the Benin material yielded no evidence to support a connection between the European trade brass and the Benin works [11–13]. It was found that the manillas available for analysis were far too impure to act as raw material [12]. No closely dated, early examples of manillas were available for analysis, studies further noted. Former analysis concluded that there is “no evidence that these manillas really did find their way into the Edo casters’ crucibles, nor do we know what they were actually made of” [12].

Other results from earlier geochemical analyses were also puzzling. A study of the lead isotope ratios in the Benin artworks revealed them to be extraordinary homogenous, at least for the bulk material analysed [5, 14]. Out of the 212 Benin Bronzes that were analysed 113 fall into what the authors describe as “unusually small field” [5] extending from ^206^Pb/^204^Pb = 18.32 to 18.45 and ^207^Pb/^204^Pb 15.59 to 15.65 or ^208^Pb/^204^Pb = 38.3 to 38.4 respectively. Significantly, lead isotope analysis is used for age determinations of a certain mineralisation in geochemistry. As lead isotope ratios do not fractionate during smelting processes, non-ferrous artefacts can be assigned to a certain mineralization when the lead isotope ratio is consistent by method of elimination. Interpretation of lead isotope ratios of leaded copper alloys such as the Benin Bronzes or the manillas is not as conclusive as for raw copper or copper ore. Adding lead to copper may result either in a mixture of lead isotopes or even in the complete overprinting of the original lead isotope ratios. Brass, as an alloy of copper and zinc frequently carries some lead that originates from the calamine ores, which generally are not lead-free. This can further complicate the picture making it unclear whether the lead isotope ratios represent the copper source, the calamine source or an intentional addition of lead. Furthermore, copper alloys with different lead isotope ratios can also be mixed for example when material is recycled resulting in indistinct LI-ratios. For the Benin Bronzes it was not yet possible to find an explanation for the lead isotope data homogeneity, as it seemed to conflict with the fact that various nations traded brass with Africa [5]. It appeared that the brass used to create the Benin Bronzes came from a singular, yet unknown, source.

Recently, R. Denk published the results of a decades long research project on manillas [15]. He notes that one of the challenges in identifying the metallic source of the Benin castings is due to the fact that stylistic differences of the manillas had not been considered. Denk developed a typological classification showing that the early manillas traded by the Portuguese, sometimes referred to in the written sources as “tacoais”, are a distinct type. Second are the so-called “Birmingham” manillas, which are stylistically different from the Portuguese “tacoais” manillas and were only produced beginning in the 18th century. A third type, are “popo” manillas that can be regarded as a stylistically transitional type between the Portuguese “tacoais” manillas and the Birmingham manillas. Subtypes also exist [15] Table 1 To identify the metal source(s) used in the production of the early Benin Bronzes it is necessary to know if the Portuguese “tacoais” manillas or the so-called Birmingham manillas are represented. These manillas have different origins, shapes, and distribution areas, as well as different metal compositions. Lead isotope analysis [16] of a single Portuguese “tacoais” manilla recovered in Cidade Velha (Cape Verde Islands) proved for the first time its European origin with copper from Banská Bystrica (Slovak Republic) and its further processing with calamine into brass in the Rhine-Meuse region.

**Table 1:** Manillas typology.

Manilla type	*tacoais*	*popo*	"*Birmingham*"
shape	Open ring with smooth transition to bulbous thickened ends. No casting seams. No shape variations.	Open ring with short set off slightly thickened ends. Nearly always casting seams. No shape variations.	Open ring. At the ends angular protruding round to heard-shaped plates. Always casting seams. Many shape variations
material	Leaded brass (up to 14%). Zn up to 25%	Leaded brass or copper alloy. Zn–content variable	"hardmetal" Cu mostly below 65%, Pb over 25%, Zn less 2%
weight	ca. 200g - 305g	ca. 100g - 150g	ca. 80g - 150g
used by	Portuguese traders	French-, Dutch-, English traders	English traders and local people in Southeast Nigeria
countries of use	Elmina (Ghana), Kingdom of Benin (Nigeria)	Ivory Coast	Southeast Nigeria (Popo, Bonny, New Calabar)
used as	trade courrency, commodity money	currency of the traders and the local people	currency of the local people
used from > to	ca. 1450—not known	1600–1914	ca. 1625–1949
production places	Germany (Rhine-Meuse region)	Nantes? Wales (Swansea)	Swansea (Wales), Bristol, Exeter and Birmingham (England)

Summarised from [15].

In the current study, documentary sources and geochemical analyses are used to demonstrate that the source of the early Portuguese “tacoais” manillas and, ultimately, the Benin Bronzes was the German Rhineland. Written sources [17] suggest that beginning in the 15th century large quantities of manillas were produced in the German Rhineland industries between Cologne and Aachen that relied on the rich occurrences of calamine (the zinc-ore necessary to produce brass) and nearby lead sources. Surviving documents [18–20] of contracts between Portuguese, Dutch, and other slave trading nations with the brass producers of this region suggest that the Benin Bronzes lead isotopic homogeneity represents a continuity of brass supply through these Rhenish industries. Herbert observed that the West Africa trade in metal goods was intricately tied to the traditional centers of copper working and brass production in Belgium and the lower Rhine, and the subsequent rise of the Antwerp merchant houses [21], but primary sources remain scarce.

The limited documentary evidence on the early Atlantic trade and bronze casting in Benin is usefully complemented by geochemical analysis on manillas dating to the period when the Benin Bronzes were produced. The use of isotopic and trace elemental analyses in establishing a connection between early Rhenish production, manillas and the Benin bronzes was initially explored in Skowronek 2021 [22]. While manillas are seldom found in archaeological excavations [23], they have been recovered from varied shipwreck contexts across the triangular trade routes between Europe, Africa, and the Americas dating between the 16th and 19th Centuries.

Here, we present the lead isotope ratios and trace element compositions of 67 manillas from five Atlantic shipwrecks and three terrestrial sites. These data are then compared with those of relevant European ores and to those of the Benin Bronzes.

## Materials and methods

### Materials

The manillas analyzed here were recovered from five shipwreck sites: Getaria, Spain; Elmina, Ghana; Cape Cod, Massachusetts; Site 35F, UK-Channel, The Scilly Islands, UK; and three terrestrial contexts: Grötö Island, Sweden; the Elmina Town Site, Ghana; and Sierra Leone. These sites are discussed in turn.

#### Getaria, Spain, 1524 (n = 19)

**Fig 1:**
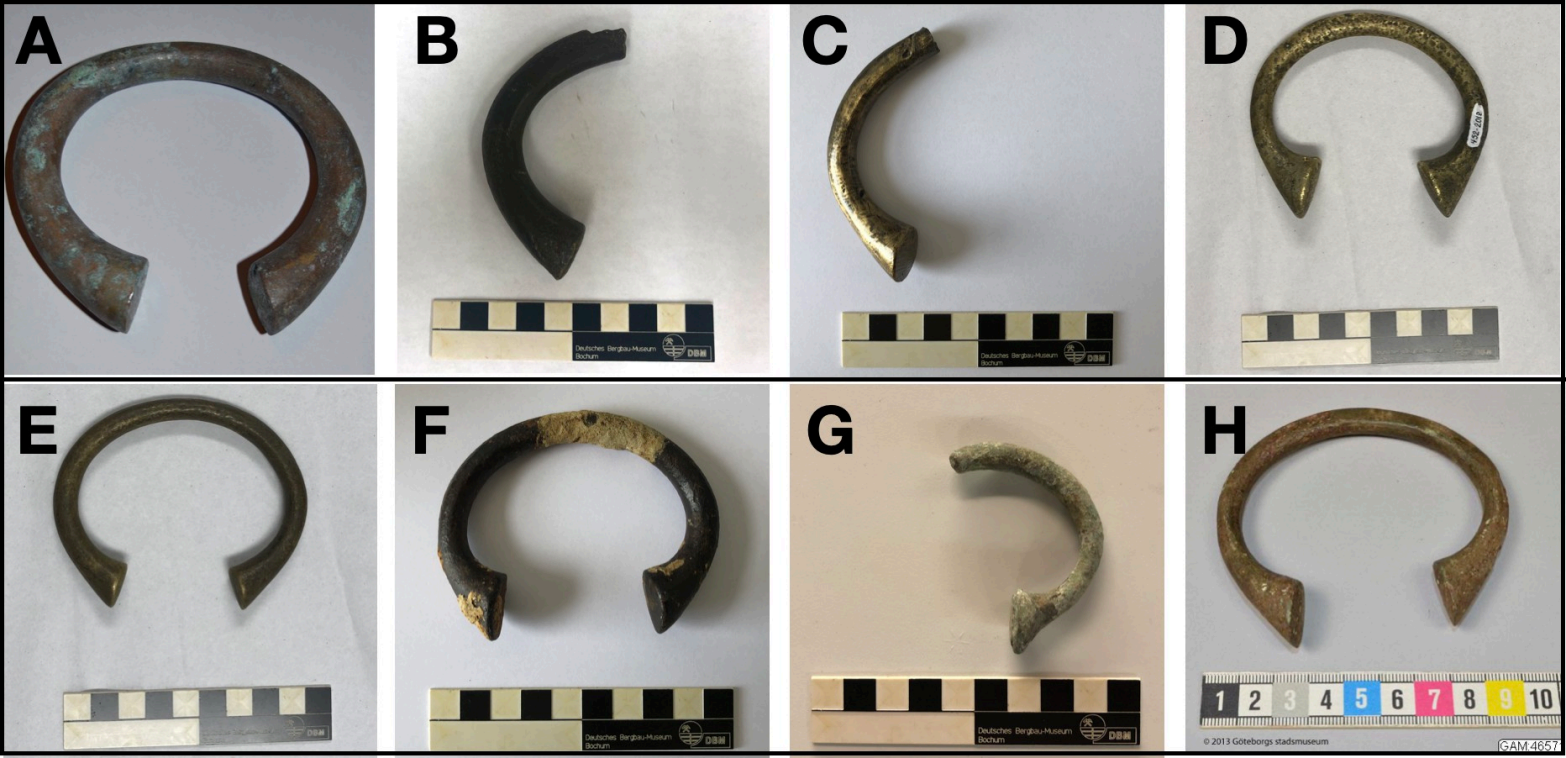
Examples of sampled manillas from the seven sites. a) Getaria, tacoais-type b) Elmina, Town Site, tacoais type c) Elmina wreck, tacoais type d-e) Cape Cod, both popo-type f) Site 35F, popo-type, g) Scilly Isles, Birmingham-type h) Grötö, popo-type. Some findspots produced various typologies. Scale is 10cm.

In 1987 divers accidently found copper ingots on the ocean floor in the bay of Getaria near San Sebastian, Spain. In the following decade the “Sociedad de Ciencias Aranzadi” carried out soundings and archaeological excavation. The results of these investigations reveal a shipwreck site of a Flemish hulk of the early 16th century. During the 1990s a variety of finds were made including metal trade goods such as copper cauldrons, copper ingots, brass basins, pins and some 313 manillas (Fig 1A). All of the last as of the stylistically similar to the early Portuguese “tacoais” manilla type described by Denk [15]. Archival work revealed that a vessel laden with all kinds of trade goods was lost during a storm most probably in 1524. It is believed that the Portuguese chartered this trading vessel in Antwerp to bring exchange goods for the Indian and African trade to Lisbon [24].

#### Elmina Town Site, Ghana, 15th-17th Century (n = 1)

Two manillas were recovered from archaeological excavations directed by C. DeCorse [23] at the African settlement of Elmina, in the Central Region of coastal Ghana. Elmina was the site of “Castelo São Jorge da Mina”, the first and largest European trade fort established in sub-Saharan Africa. The fort was the Portuguese headquarters in West Africa from its founding in 1482 to its capture by the Dutch in 1637. Many of the finds at the site can be closely dated based on European trade materials. The manilla sampled was recovered from a pre-19th century stratigraphic context, likely dating between the late 15th century and the early 17th century. Only half of the manilla is present, but stylistically it is the Portuguese “tacoais” type (Fig 1B). The flared end of the manilla is battered indicating reuse as a hammer.

#### Elmina Wreck, Ghana, 1647 (n = 10)

The discovery of the Elmina Wreck resulted from the first maritime archaeology research project conducted in Ghana. Research specifically focused on identifying wreck sites off the town of Elmina. Side-scan sonar survey and diver investigations resulted in the discovery of a mid-17th century shipwreck, which archaeological and archival research suggests may be the Dutch West India Company vessel Groeningen that sank after arriving at Elmina on a trading voyage in 1647. Multiple radiocarbon dates from the ship’s hull correlate well with this date. The site lies approximately 2.4 km southeast of Elmina in approximately 12 meters water, and is characterized by a mass of trade goods including brass and pewter basins, brass manillas, lead rolls, trade beads, pins, cowrie shells, as well as large iron cannons. Out of more than 600 manillas 44 specimen resembling the “tacoais” type (Fig 1C) were recovered. Artifacts from the wreck site provide insights into the commodities involved in exchange between Africans and Europeans at a time when the trade in slaves was becoming increasingly important [25].

#### Cape Cod, Massachusetts, United States, 1717 (n = 20)

In the 2018 and 2019 field seasons, while investigating a previously unexplored section of the Whydah Gally wreck site (WLF-HA-1), off Cape Cod, Massachusetts, United State, archaeological field crew discovered and retrieved a collection of 768 manillas. Although located several hundred feet south and inshore from the bulk of wreckage materials their association with the Whydah is confirmed by the presence of other diagnostic artifacts, most notably Spanish colonial currency, but also a small cannon, copper rods, pewter wares, gold, and glass bottle fragments in the same vicinity. Consisting of two distinct morphological types, one with more flared ends (Fig 1D) and the other with more constricted ends (Fig 1E) both types can be referred to as “popo” manillas. Ten manillas of each type were chosen for sampling. These manillas are assumed to be leftovers from the Whydah Gally’s collection of English trade goods destined for the West African slave trade. To date, over 200,000 artifacts have been recovered from the Whydah, and it is anticipated that more manillas will be found as excavations continue [26].

#### Site 35F, English Channel, 17th-18th Century (n = 1)

In 2002 French company Comex (Compagnie maritime d’expertises) identified a wreck at a depth of 110 meters in the western approach to the English Channel. The wreck was extensively excavated, without a publication. The remains were relocated in 2005 by Odyssey Marine Exploration of Florida and further surveys and excavations were conducted in 2006, 2008, and 2009. Side-scan sonar and on-site observations confirmed the wreck had been extensively impacted by fishing trawlers, including destruction by scallop dredges. A selection of 58 diagnostic artifacts were recovered, ranging from ballast stones and patches of lead hull sheathing to cannonballs, elephant tusks, and nine manillas [27]. The combination of elephant tusks, manillas, and a stack of copper basins, as well as furring in the hull construction (a double layer of outer hull strakes), all typify a ship trading with West Africa. The mathematical formula of an inch used in a wooden folding rule and glass bottle types, point to an English nationality for the vessel and a circa 1660 to 1700 age, perhaps more closely dating between 1672 and 1685 [27, 28]. At this time, the Royal African Company (RAC) held a royal monopoly over the English trade with West Africa. The wreck is identified as a RAC merchant vessel that had travelled to the Gold Coast, possibly dropped off enslaved Africans in the Caribbean, and was close to home in London, likely with a cargo of sugar, when it sank. Eric Tate provided one of the manillas for analysis. It is stylistically comparable to the “popo” type (Fig 1F).

#### The Scilly Islands, United Kingdom, 1843 (n = 11)

In 1972 divers found the remains of a 19th century trading vessel on the western rocks of the Scilly Islands, United Kingdom. A large volume of finds recovered from the wreck include blue and white glass trade beads and some three tons of manillas piled 4 feet high. It is likely that the wreck is the “Douro”, a 200-ton snow (square-rigged vessel with two masts) built in Sunderland, England in 1839, and owned by Paull & Co. The ship sailed from Oporto, Portugal for Liverpool on 17 January 1843, outbound for West Africa, and sank on 26 January after colliding with the Round Rock off the western Isles of Scilly. An account published in the “Royal Cornwall Gazette”, 3 February 1843, includes Lloyd’s list entry No. 9017 for 2 February 1843. The entry describes the cargo as consisting of baled goods, “armoury and brass stops”. Underwater reconnaissance proved the “brass strops” to be the largest consignment of manilla bracelets encountered on a shipwreck worldwide [29]. Stylistically the manillas are of the “Birmingham” type (Fig 1G). Richard Larn donated the manillas to the Shipwreck Treasure Museum in Charlestown, Cornwall where 10 of them were sampled. Another one was provided by Tony Coverdale of the Saltford Brass Mill project.

#### Grötö Island, Sweden, date unknown (n = 4)

In 1936 during construction work on the island of Grötö, near Gothenburg on the west coast of Sweden eight copper-alloy rings were discovered under a boulder close to the shore. Ernst Nelsson, on whose property the rings were found, brought them to the Gothenburg Museum. It was first thought that they represented a baltic import from the Merovignian period [30]. They were correctly identified as manillas by British Museum archaeologist W. Fagg in 1957. The manillas are of the”popo” style (Fig 1H). It is unknown how they ended up on the remote island of Grötö. Grötö is however part of Kalvsund, which has been a frequented anchorage since medieval times.

#### Sierra Leone, date unknown (n = 1)

In 1992, C. DeCorse noted a single manilla in a collection of archaeological materials for sale in the tourist market in Freetown, Sierra Leone. The artifact included fragments of local ceramics, several brass bracelets of local or West African manufacture, and a single manilla. The artifacts had no provenience other than that they were said to come from a single context in southern, coastal Sierra Leone, possibly Sherbro island. The appearance of the materials were consistent with this attribution. They were encrusted with soil and the brass materials extensively oxidized. Stylistically, the manilla is of the early Portuguese “tacoais” type. A sample of the manilla was provided by C. DeCorse.

### Methods

#### Sampling

In order to see any scatter of the analytical data sample selection aimed at the highest amount of individual samples possible. However, as most finds were either under state, institutional or private ownership availability for sampling depended on the allowance of those owners. In most cases, damaged pieces were chosen for the invasive sampling.

Manillas were sampled using High-Speed-Steel (HSS) drill bits of 1.0 or 1.5mm diameter attached to a portable Dremel tool. Drill bits were treated with isopropanol before sampling and were discarded after each object. Corrosion layers were carefully removed by surface drilling until fresh metal was visible. Approximately 50mg of metal swarf were obtained by drilling a single approx. 0.5 cm deep hole into each object.

#### SC-ICP-MS trace element analysis

Metal samples have been dissolved with a 1:1 mixture of 1.5 ml each 7 n HNO3 and 6 n HCL, afterwards diluted with ultrapure water to a concentration of c. 1000 mg/L. Chemical analyses have been performed with an HR-ICP-MS (Thermo Fisher Scientific ELEMENT XR). Quantification was done with external calibration. For main and minor elements (Cu, Zn, Pb, Sn, Sb, As), sample solutions have been diluted 1:100, for traces 1:10 with 5% HNO3. The analyses have been carried out with a FAST SC-system, ST 5532 PFA μ-FLOW nebulizer, Peltier-cooled PFA spray chamber and 1.8 mm sapphire injector in triple detector mode at all three different mass resolutions (m/Δm) depending on the elements of interest. Measurements have been controlled with compatible standard materials (copper metal standard BAM 376 (Bundesanstalt für Materialforschung, Berlin), brass metal standard BAM 223 and tin bronze metal standard BRONZE C (British Chemical Standards, Middlesbrough, UK)). Relative standard deviation for trace elements varied between 0.6 and 5%, for main elements between 0.6 and 2%, depending on the level of concentration in the sample solution.

#### MC-ICP-MS Lead isotope analysis

Lead (Pb) isotope ratios were measured using MC-ICP-MS with a Neptune XT (Thermo Fisher Bremen) at the research laboratory of the Deutsches Bergbau-Museum in Bochum. For samples with less than 0.5 wt.-% lead content, classical HBr ion exchange chromatography was applied using AG1-X8 resin (Bio-Rad Laboratories, Inc.). Pb isotopes were measured using wet plasma conditions. For mass bias correction, 200 ppb lead solutions were doped with 50 ppb thallium (NIST SRM 997). 202Hg was recorded for interference correction. Before and after every five samples, the reference material NIST SRM 981 was measured in the same way to compensate drift and ensure accuracy. The recommended values by Taylor et al. [31] are used for final normalization of the samples. The methodology leads to an external precision better than 0.005 percent for 204Pb normalized ratios and 0.002 percent for 206Pb normalized ratios.

## Results

### Chemical composition

The manillas analysed here are chemically different from each other. Although most manillas analysed here (S1 Table) are brasses or leaded brasses, sometimes with small amounts of tin, a few specimens from Grötö and the Scilly Islands are leaded copper with little or no zinc. Among the brass manillas, the amounts of lead vary between 1 and 14 wt.-%, while amounts of zinc range between 15 and 25 wt.-%. Nineteenth century British manillas from the Scilly Islands and the specimen from Grötö Island, Sweden have lead amounts over 20 wt.-%.

Antimony is by far the greatest impurity among trace elements in the manillas studied. The 19th century manillas contain up to 8.6 wt.-% of antimony, while pre-19th century specimens range between 0.01 and 4.4 wt.-% though most manillas are below 1 wt-%. Nickel is present in amounts of up to 0.7 wt.-% but the bulk of manillas range around 0.2 wt.-%. The amount of nickel is comparatively low in late manillas (0.05 wt.%). Amounts of arsenic are worth mentioning too as this element can be present in up to 0.6 wt.-% though most manillas have arsenic amounts under 0.2 wt.-%. Iron can also be present up to 1 wt.-% but most manillas have amounts under 0.5 wt.-%. All other trace elements are under or around 0.1 wt.-%.

### Lead isotope analysis

The majority of manillas have lead isotope ratios between 206Pb/204Pb = 18.29 to 18.43 and 207Pb/204Pb = 15.61–15.63 or 208Pb/204Pb 38.3–38.45 respectively. 19th century manillas and those of early British production (late 17th-early 18th Century) show a wider range, extending from 206Pb/204Pb 18.17 to 18.53 and 207Pb/204Pb = 15.61–15.71 or 208Pb/ 204Pb 38.1–38.8 respectively S1 Table.

## Discussion

Manilla production strongly depended on African demand. African traders are said to have been extraordinarily particular on which manillas they would accept [8, 21, 23, 32], meeting customer wishes was of primary importance. A contract between the German merchant family of Fugger and the Portuguese King dating to 1548 clearly identifies two types of manillas: one kind for the “de la Mina” trade and the other for the “Guine” trade [33]. In this context, de la Mina is referring to an area roughly corresponding to the coast of modern Ghana, while Guine [Guinea] indicates the broader region of sub-Saharan West Africa [9, 21]. The contract is very detailed and stipulates that the pieces produced should match the models provided, be of comparable quality, and the weights be 250g for the Guinea type and 312g for the Mina type.

The presence of manillas of varied composition, such as those made of tin and lead found in the 1990s near Vigo in Spain [22], attests to the fact that European producers experimented with varied compositions early on. While the composition of the earliest manillas from the 16th century Getaria wreck reflect the use of a copper relatively low in trace elements, the specimens from the mid-17th century onwards often have high amounts of antimony; an indication that a rather impure copper was soon used in manilla manufacture. Copper of similar composition was used for making cauldrons; the so-called caldarium copper that was only suitable for casting [34]. This kind of copper would have been much cheaper than the stronger refined copper, used for forging, wiredrawing, and the production of hollow wares.

**Fig 2:**
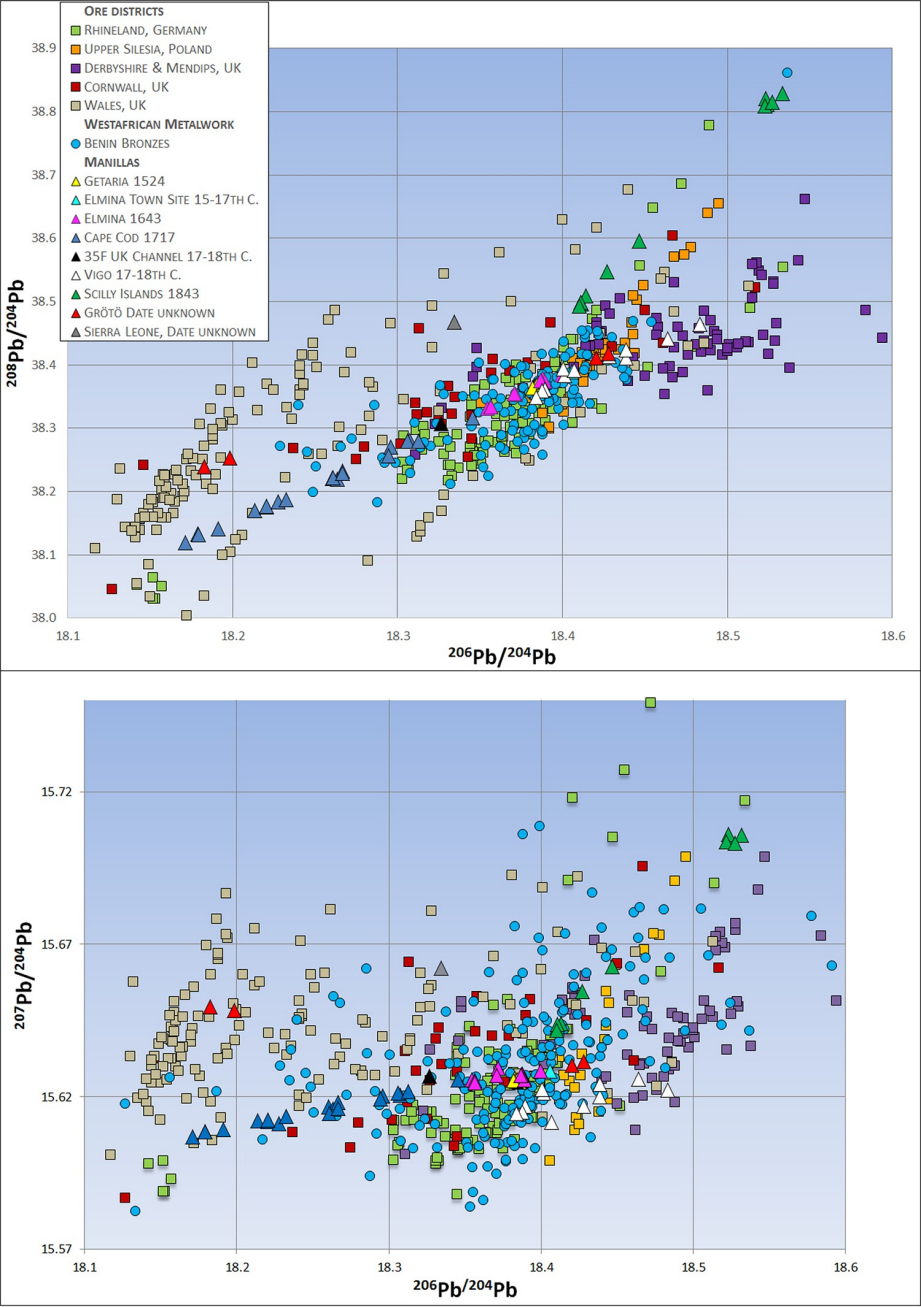
Lead isotope ratios 206Pb/204Pb against 207Pb/204Pb and 206Pb/204Pb against 208Pb/204Pb of various ores vs. those of Benin Bronzes and manillas of different origin and age. Only those manillas that match with the Rhenish lead-zinc ores also match the Benin Bronzes. Manillas from the 18th century onwards can be related to some of the Benin Bronzes but do not fit to the bulk cluster of the famous artworks. This bulk cluster is in good accordance with the Rhenish ores. 2sigma errors are below the size of the symbols. Data for Rhenish ores [37–43], Upper Silesia [44–46], UK Ores [47], Benin Bronzes [5], Vigo manillas [22].

Also notable is the high lead content that almost all manillas have in common. Lead in brass results in an easy flowing alloy, and reduces the porosity, making the alloy more suitable for casting [35]. The high lead amount in early manillas can be interpreted as an intentional addition to produce an easy flowing casting brass. It is astonishing that early 16th century slave traders apparently recognized African needs for manillas of specific composition. However, when trying to provenance the raw materials used for the making of the leaded manillas, using lead isotopy will only point to the source of the lead. The manillas from Cape Cod and Sierra Leone represent an exception having lead amounts of around 1 wt.-% S1 Table. Here the lead isotope ratios might either represent a mixture of the copper and lead-zinc ores lead isotope signature or the copper source itself, if the latter brought in some lead. The latter seems to be the case here as the lead isotope ratios of the Cape Cod manillas have good accordance with those of Cornish and Welsh ores (Fig 2).

**Fig 3:**
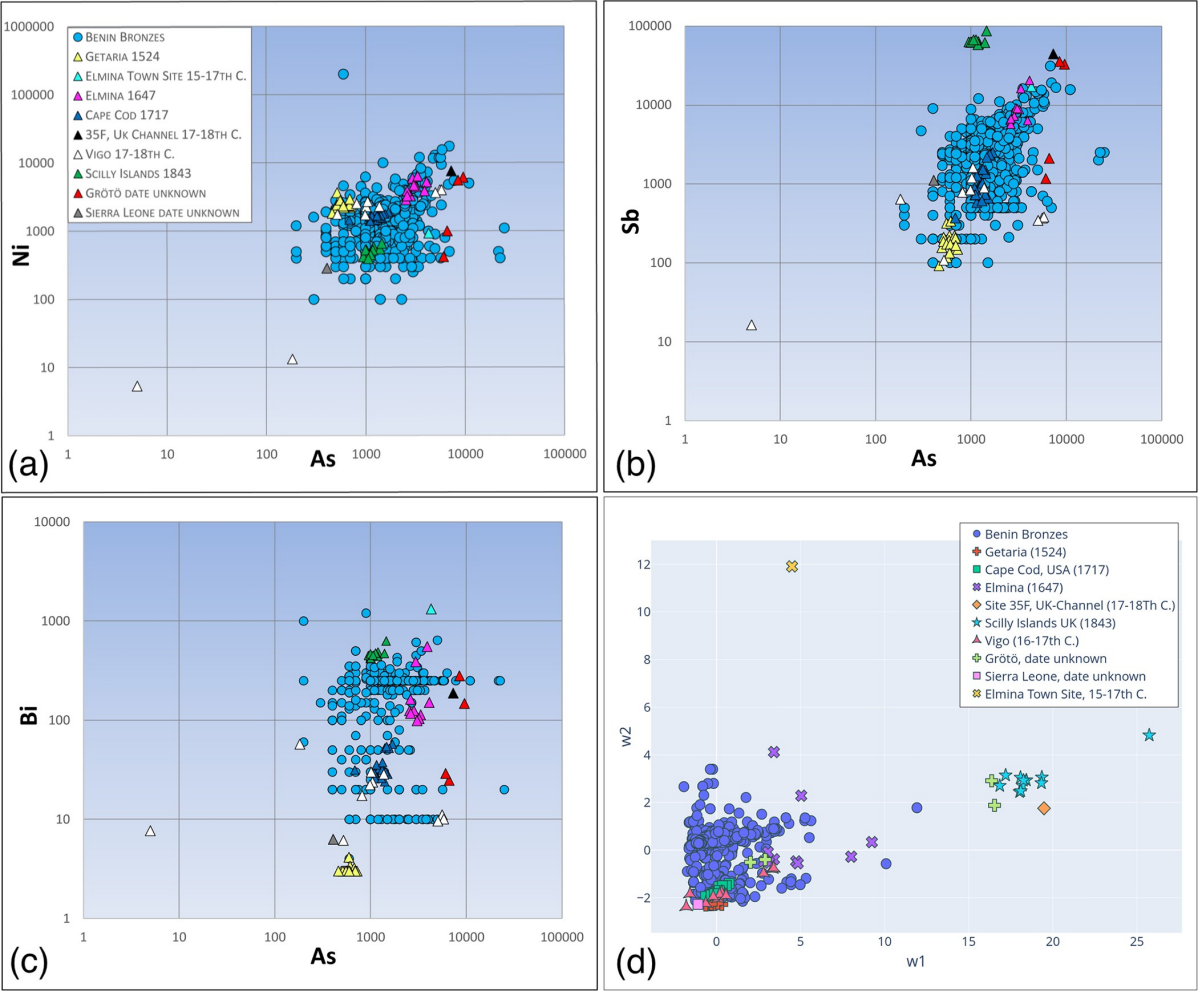
Binary logarithmic plots (a-c) and PCA (d) of trace elements Sb, Ni, Bi, and As given in ppm of Benin Bronzes and manillas. The variation in trace element content is greater in the Benin bronzes than within the individual manillas groups. The different manillas groups have distinct trace elemental patterns especially concerning amounts of Sb, As and Bi. Note that with the Benin Bronzes Bi had a detection limit of 250ppm or 10ppm depending on the analytical instrument used for their measurements. Data sources for the Benin Bronzes [3], Vigo Manillas [22].

**Fig 4:**
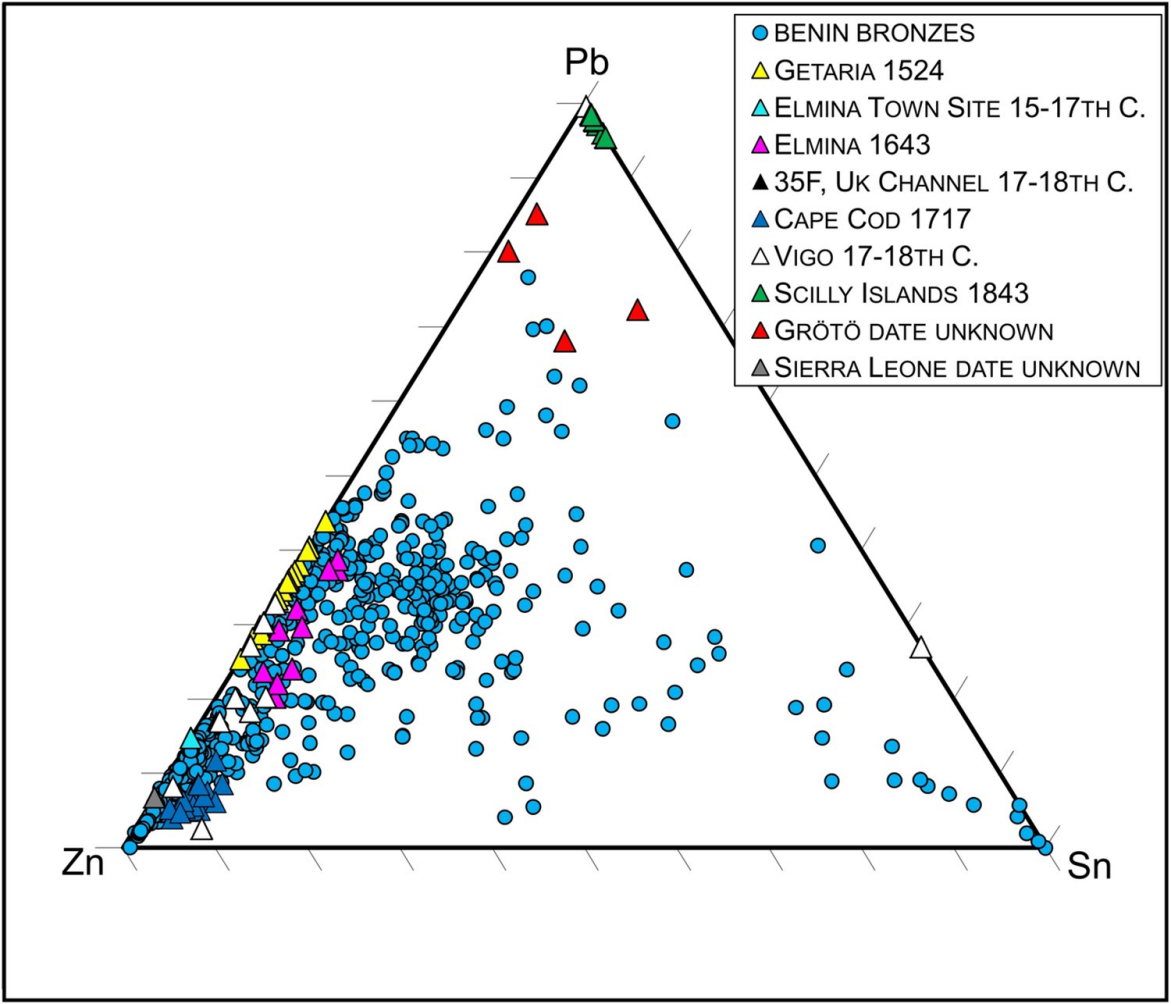
Ternary diagram for main alloying elements Pb, Sn and Zn of both Benin Bronzes and manillas. Most of the Benin Bronzes and manillas are brasses or leaded brasses. Late British manillas and those from Sweden are leaded coppers. A lot of the Benin Bronzes have higher amounts of Sn probably indicating an admixture, possibly bronze. Note the two outliers from Vigo which are no copper alloys but were made of lead and a lead-tin alloy respectively. Data sources for the Benin Bronzes [3], Vigo manillas [22].

African consumption is plainly revealed when the lead isotopes of the manillas are compared to those of various Benin Bronzes (Fig 2). Pre-18th century manillas share strong isotopic similarities with Benin’s famous artworks. Trace elements such as antimony, arsenic, nickel and bismuth are not as similar as the lead isotope data (Fig 3). The greater data derivation suggests that manillas were added to older brass or bronze scrap pieces to produce the Benin works, an idea proposed earlier [11, 12]. Judging from the amounts of main alloying elements Zn, Pb and Sn this appears to be the case, at least for those of the Benin Bronzes that have higher amounts of Sn than the manillas (Fig 4). Bronze as an alloy of copper and tin would also bring in trace elements, resulting in a mixture hard to interpret. Recent research on copper ingots of the Early Modern era has revealed that depending on the kind of copper ore used the trace elemental signature, to be exact, amounts of antimony, arsenic, nickel and bismuth are characteristic. For example, while the fahlore copper of the Alpine and Slovakian deposits results in a copper high in Sb and As, the central German deposits in the Mansfeld area produced a copper high in Ni and extremely low in Bi [22]. As many different copper sources were used for the making of the manillas (see discussion below for potential sources) and were possibly mixed already during their fabrication, trace elemental interpretation must be handled with care.

From the mid-17^th^ century onwards, manillas frequently carry some tin. This may be due to the beginning of Cornish copper mining, as it is known that the Cornish copper ores are related to tin, its presence posed a refinery problem [36]. In fact, all manillas of British production analysed here carry some amounts of tin.

For the isotopic signature, the high lead content of the manillas may have overprinted any other lead isotope ratios of the other components used for the making of the Benin Bronzes. Thus, there is little chance of identifying the source of the other components. However, it should be noted that the 9-10th century bronzes from Igbo-Ukwu, Nigeria testify to use of distinct bronze in casting long before the opening of the Atlantic trade [4].

The lead isotope ratios of early manillas from 16th-17th century shipwrecks share stronger similarities with those of the Benin Bronzes then those of later manillas (Fig 2). The early manillas (the Getaria and Elmina wrecks, the Elmina Town Site, and some of the Vigo manillas) also match well isotopically with the lead-zinc ores of the German Rhineland making it likely that they were produced by the well-established brass industry of that particular region (Fig 2). Other lead-zinc occurrences such as the Mendip Hills and Derbyshire in the United Kingdom, or in Upper Silesia poorly match either the early manillas or the Benin Bronzes. In addition to local occurrences of zinc and lead ores, the Rhenish industry imported copper from a variety of localities including Norway, Sweden, central Germany, and Slovakia via the seaway from Baltic ports [48]. Copper sourced from these various localities may explain the heterogeneity of trace elements among manillas (Fig 3). As pointed out above, copper mining districts in Europe produced copper with characteristic trace elemental patterns. While comparisons between trace elements of ores and metals has been proven to be ineffective due to the fractionation of most elements during smelting processes [49] trace elements of various copper metal can still be compared to each other. The Getaria manillas have low Sb, As and especially low Bi amounts (Fig 3) characteristic for the copper of the Mansfeld area in central Germany [22]. As copper from Mansfeld was frequently used in Rhenish brass production [48] the former is a likely source for the copper in the Getaria manillas. Elevated amounts of antimony (more than 1000ppm) is common for all other manillas studied here (Fig 3) and their presence may point to the use of fahlores [50]. Those ores occur in Slovakia, in Alpine deposits but also in Cornwall [34] the latter being the likely source from 1650 onwards. As pointed out above, the Fugger family and other merchant firms had a lease on the copper mines near Banská Bystrica in Slovakia and may have brought this antimony-rich fahlore copper in tons to the Rhenish brass producers. One should not forget that contrary to earlier time periods material flows were on a global scale. The Dutch already brought copper from as far away as Japan to Amsterdam early in the 17^th^ Century [51]. The Grötö manillas are an example for the possible use of various raw materials. Two of them are high in Sb, As, Ni and Bi (Fig 3) the other two have lower amounts in these particular elements. The two manillas with high amounts of trace elements isotopically plot with the Silesian Pb-Zn ores while the other group plot with the Welsh lead ores (Fig 2). This possibly indicates that those manillas might have been produced in either the Kingdom of Denmark or Sweden by buying those raw materials at the trade fairs or markets of the time. The Vigo wreck, on the other hand, may represent a vessel coming back from West Africa to Spain bringing back manillas and possible other cargo. This explains the heterogeneity of their trace elements and lead isotope ratios, as they probably have not been produced at the same time nor the same place. Some of them carry the typical lead isotope signature of the Rhenish lead-zinc ores (Fig 2) and trace elemental patterns comparable to central European copper but others have trace elements more similar to the Cape Cod manillas (Fig 3), probably representing Cornish copper. The lead isotope ratios of these manillas have more similarities with British lead occurrences (Fig 2) One of the Vigo manillas is made of lead another of a tin-lead alloy. These manillas represent outliers in all trace and main element plots. Their lead isotope ratios are consistent with ores from Derbyshire probably the biggest lead mining district of this time-period (Fig 2).

**Fig 5:**
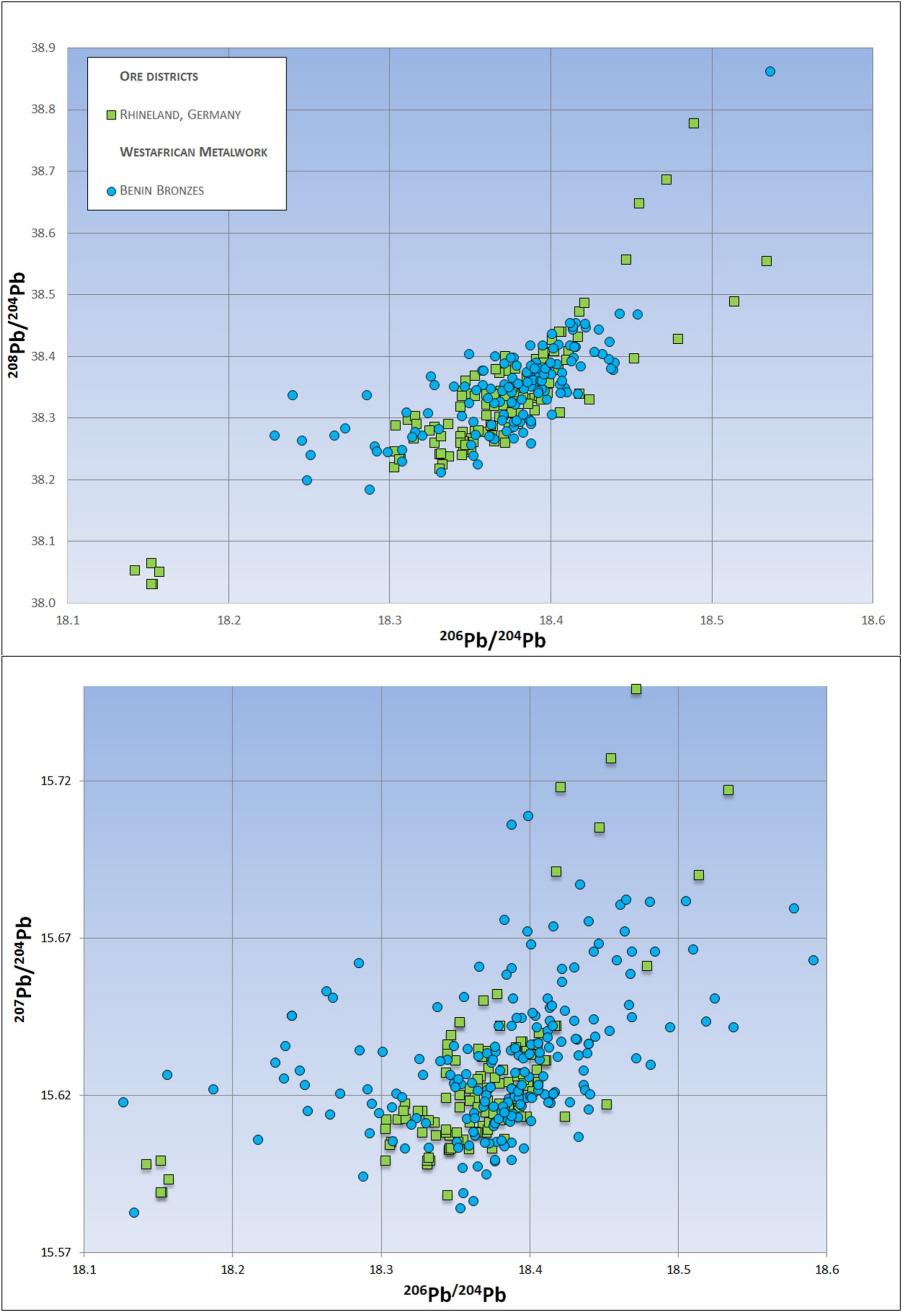
Lead isotope ratios 206Pb/204Pb against 207Pb/204Pb and 206Pb/204Pb against 208Pb/204Pb for Benin Bronzes and Rhenish lead-zinc ores. The lead isotope ratios of the Benin Bronzes have striking similarity with the lead-zinc ores of the German Rhineland. Data for Rhenish ores [37–43], Benin Bronzes [5].

The most striking similarities in the lead isotope ratios are between the Rhenish lead-zinc ores and those of the Benin Bronzes (Fig 5). The lead isotope data progression is remarkably uniform, indicating the mass-export of metals made from Rhenish sources. In this regard, it should be noted that manillas were not the only product of this industry that went to West Africa. Finger thick brass rods known as “Guinea Rods”, and various hollow ware such as brass basins, pots, and kettles were traded almost as extensively as manillas [7, 21] along the entire West African coast [52]. Analysis of these may provide further insights into the making of the Benin artworks.

In Europe, calamine and lead might also have been traded to other localities where copper was available to produce brass. But as much of the zinc was lost in the cementation process it required twice as much calamine then copper only to produce a common brass with 70 wt.% copper and approx. 30 wt.% zinc [53]. This would make it less economical to trade the calamine to places where copper was readily available. Thus, brass producers located in places where calamine occurred [54]. Consequently, in the case presented here, the lead isotope data does not only reflect the raw materials used but also where the manillas were most likely produced.

## Conclusion

The work presented affords new insights into the early Atlantic trade, African consumption and production of European metal goods, and the chronology of both European and African castings.

Although the importance of European brass, including the potential role of Rhenish sources, in African casting industries has long been recognized, this study definitively identifies the Rhineland as the principal source of manillas at the opening of the Portuguese trade. Millions of these artifacts were sent to West Africa where they likely provided the major, virtually the only, source of brass for West African casters between the 15th and the 18th centuries, including serving as the principal metal source of the Benin Bronzes. However, the difference in trace elemental patterns between manillas and Benin Bronzes does not allow postulating that they have been the only source.

While early Portuguese “tacoais” type manillas were an exclusive product of the Rhenish industries, other European manufacturers entered this profitable production sector in the 18th century. These later products are, however, distinct both typologically and metallurgically from the earlier Portuguese manillas. The trace element and lead isotope ratio data presented here indicate that these later manufactures from England and, perhaps, Scandinavia were fabricated using different copper alloys that did not find their way into the crucibles of the Edo casters that produced the Benin Bronzes.

Manillas had no purpose in European societies: they were a product specifically produced for the African trade and it is clear from documentary sources that Africans were selective in the products they accepted. Edo metalsmiths were likely well aware of the better casting qualities of the Portuguese “tacoais” type manillas, and these were subsequently demanded in trade. Ongoing research may afford additional insights into other West African casting traditions.

## Supporting information

S1 Table Analytical dataset. This dataset contains the analytical data obtained by ICP-MS analysis as shown in Figs 2–4 in the main manuscript text and as described in the materials and methods section. Chemical elements are given in weight percent (wt.-%). The isotope ratios error 2SD means two times standard deviation.
(XLSX)
